# Heat shock protein 60 stimulates the migration of vascular smooth muscle cells via Toll-like receptor 4 and ERK MAPK activation

**DOI:** 10.1038/srep15352

**Published:** 2015-10-19

**Authors:** Ying Zhao, Chenxu Zhang, Xuge Wei, Pei Li, Ying Cui, Yuanhua Qin, Xiaoqing Wei, Minli Jin, Kazuhiro Kohama, Ying Gao

**Affiliations:** 1 Liaoning Provincial Core Lab of Medical Molecular Biology, Dalian Medical University, Dalian, 116044, China; 2 Department of Biochemistry and Molecular Biology, Dalian Medical University, Dalian, 116044, China; 3 Research Institute of Pharmaceutical Sciences, Musashino University, Nishitokyo, Tokyo, 2028585, Japan

## Abstract

Accumulating evidence indicates that heat shock protein (HSP) 60 is strongly associated with the pathology of atherosclerosis (AS). However, the precise mechanisms by which HSP60 promotes atherosclerosis remain unclear. In the present study, we found that HSP60 mRNA and protein expression levels in the thoracic aorta are enhanced not only in a mouse model of AS but also in high-fat diet (HFD) mice. HSP60 expression and secretion was activated by platelet-derived growth factor-BB (PDGF-BB) and interleukin (IL)-8 in both human umbilical vein endothelial cells (HUVECs) and vascular smooth muscle cells (VSMCs). HSP60 was found to induce VSMC migration, and exposure to HSP60 activated ERK MAPK signaling. U0126, an inhibitor of ERK, reduced VSMC migration. The HSP60-stimulated VSMCs were found to express TLR4 mRNA but not TLR2 mRNA. Knockdown of TLR4 by siRNA reduced HSP60-induced VSMC migration and HSP60-induced ERK activation. Finally, HSP60 induced IL-8 secretion in VSMCs. Together these results suggest that HSP60 is involved in the stimulation of VSMC migration, via TLR4 and ERK MAPK activation. Meanwhile, activation of HSP60 is one of the most powerful methods of sending a ‘danger signal’ to the immune system to generate IL-8, which assists in the management of an infection or disease.

Atherosclerosis is a primary cause of morbidity and mortality worldwide. It is a multifactorial disease for which a number of different pathogenic mechanisms have been proposed. Over the past decade, it has becoming increasingly clear that an infection may be an important initiating component of the atherogenic process^1^,^2^. Heat shock protein 60 (HSP60) is structurally highly conserved and abundantly expressed by prokaryotic and eukaryotic cells under stress conditions. HSP60 is encoded in the nucleus and expressed in the mitochondria. Under conditions of stress, HSP60 is transported to the cytosol and subsequently appears on the cell surface where it acts as a “danger signal” for innate and adaptive immunity^3^,^4^. Accumulating evidence indicates that HSP60 is strongly associated with atherosclerosis. Several epidemiological studies have shown a positive association between titers of antibody to HSP60 and the extent of atherosclerosis^5^,^6^,^7^,^8^. Xiao and colleagues hypothesized that soluble HSP60 (sHSP60) is involved in activating proinflammatory processes associated with early vessel pathology. They provided the first prospective data confirming an association between high levels of sHSP60 and early carotid atherosclerosis^9^. Several animal experiments have shown that plasma titers of antibody to HSP60 are strongly associated with lesion formation in cholesterol-fed rabbits and increased expression of HSP60 on the endothelium during atherogenesis^10^,^11^,^12^,^13^. It is also known that dysregulated proliferation and migration of vascular cells in response to environmental stimuli play a key role in the development of atherosclerosis. Recently, *in vitro* experiments have shown that HSP60 can activate the proliferation of VSMCs^14^,^15^,^16^,^17^. However, the precise mechanism by which HSP60 promotes atherosclerosis remains unclear.

In the present study, *in vivo*, we tested the expression and distribution of HSP60 in the thoracic aorta in an atherosclerotic (AS) mouse model or a high-fat diet (HFD) mouse model. *In vitro*, we observed changes in the expression and secretion of HSP60 when HUVECs and VSMCs were exposed to stress and examined whether HSP60 stimulates the migration of VSMCs. Finally, we investigated the possible signaling pathways involved in HSP60-regulated VSMC migration.

## Results

### Expression and distribution of HSP60 in blood vessel walls of AS and HFD mouse models

**Figure 1:**
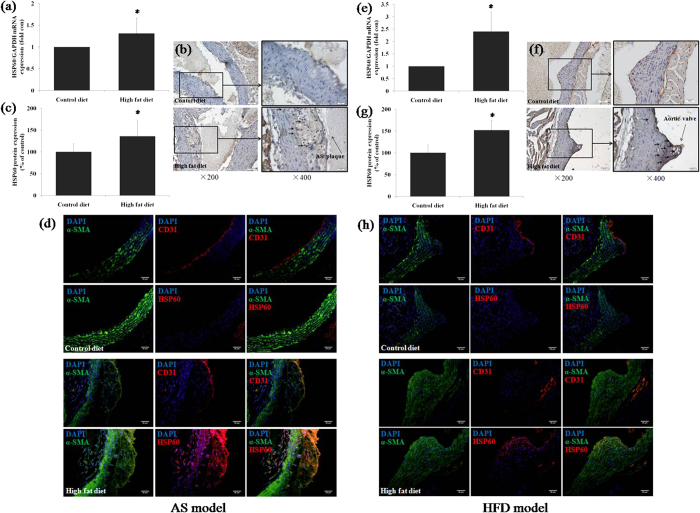
Expression and distribution of HSP60 in blood vessel walls in AS and HFD mouse models. Aortas were obtained for RNA preparation, and real-time RT-PCR analyses were performed in the AS model (**a**) and HFD model (**e**). The data are expressed as the mean ± SD (*n *= 3). *p < 0.05 compared with the control diet group. The aortas were excised for immunohistochemical (**b**,**f**) and immunofluorescence staining (**d**,**h**). Arrows indicate staining of HSP60 in AS (**b**) and HFD (**f**) models. The HSP60-positivity of cells was quantified (**c**,**g**). *p<0.05 compared with control diet group.

Thoracic aortas were obtained for RNA isolation and detection of HSP60 gene expression. The expression of HSP60 mRNA was found to be upregulated in both AS and HFD mice (Fig. 1a,e). In AS mice, HSP60 protein expression in atherosclerotic plaques was enhanced (Fig. 1b,c), and the same phenomenon was observed in the HFD mice. We know that the early stages of atherosclerosis formation appeared in the aortic root, and our data show that HSP60 staining was significantly increased in the aortas of HFD mice (Fig. 1f,g). Consistent results were obtained by immunofluorescence staining (Fig. 1d,h). We also found that HSP60 expression was mainly distribution in the smooth muscle cell layer of blood vessel walls.

### Expression and secretion of HSP60 in blood vessel wall cells

**Figure 2:**
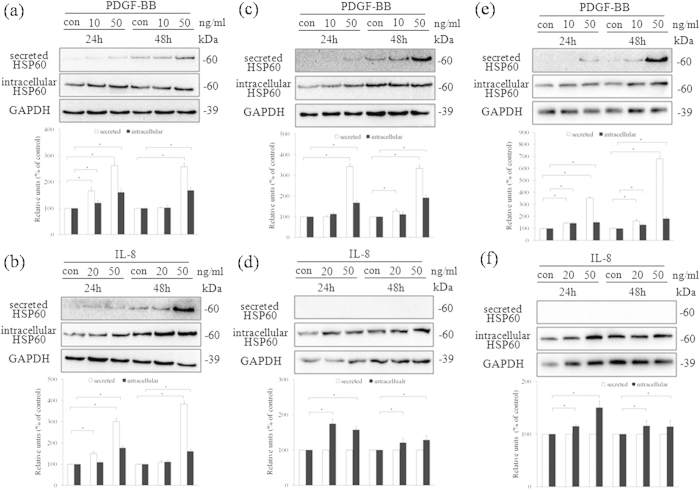
Expression and secretion of HSP60 in HUVECs and VSMCs. HUVECs (**a**,**b**), A7r5 cells (**c**,**d**), and AoSMCs (**e**,**f**) were treated with PDGF-BB (**a**,**c**,**e**) or IL-8 (**b**,**d**,**f**) at the indicated concentrations and times. The cell conditioned media and lysates were analyzed by western blotting using anti-HSP60 antibody. Equal protein loading was confirmed with antibody against GAPDH. The results were quantified and plotted in the bar graphs and are expressed as the means ± SD (*n *= 3). *p < 0.05 compared with the control group. In (**a-f**), the full length blots were presented in Supplementary Figure S2 (**a-f**).

Under physiological conditions, human vascular endothelial cells do not express HSP60 on their surface^3^,^4^. However, when endothelial cells are exposed to stress caused by atherosclerosis risk factors, simultaneous expression of HSP60 and adhesion molecules occurs. We have previously reported that PDGF-BB and IL-8 are atherosclerosis risk factors and are able to induce the migration and proliferation of VSMCs^18^,^19^,^20^. However, induction of endogenous HSP60 expression by PDGF-BB or IL-8 in cells of the blood vessel wall requires confirmation. *In vitro*, our data showed that PDGF-BB or IL-8 induced the secretion and expression of HSP60 in a dose- and time-dependent manner in the conditioned media and lysate of HUVECs (Fig. 2a,b). In addition, PDGF-BB induced the secretion and expression of HSP60 in a dose- and time-dependent manner in the conditioned media and lysate of A7r5 cells (Fig. 2c) and AoSMCs (Fig. 2e). Finally, IL-8 induced the expression of HSP60 in lysates from A7r5 cells (Fig. 2d) and AoSMCs (Fig. 2f), but not in the conditioned media from these cells.

### HSP60-induced migration of VSMCs

**Figure 3:**
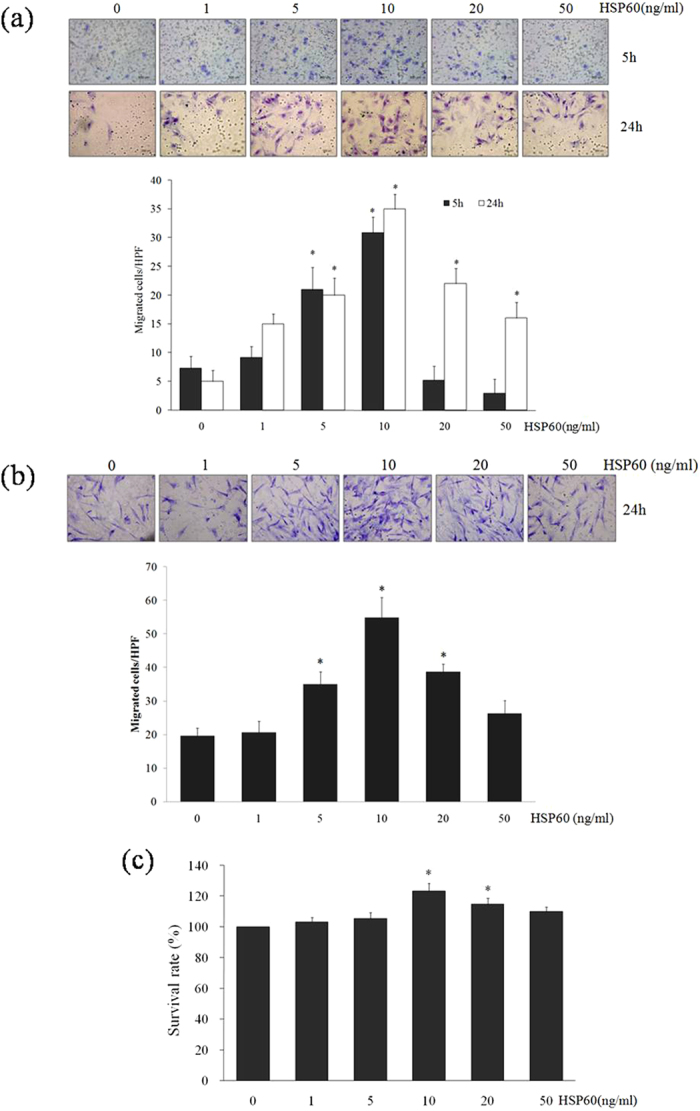
HSP60-induced migration of VSMCs. Migrated A7r5 cells (**a**) and AoSMCs (**b**) are shown with a purple color after exposure to the indicated concentrations of HSP60 for the indicated amounts of time in the Boyden chamber assay (top panels). The number of migrated cells was quantified by microscopy (HPF × 200, bottom panels). Cellular viability was assessed using the MTT assay. The results were obtained as a percentage cell viability (OD of the experiment sample/OD of the control) (**c**). All results are expressed as the means ± SD (*n *= 3). *p < 0.05 compared with control.

The Boyden chamber-based migration assay results revealed that migration of VSMCs was induced by HSP60. The greatest induction of migration was observed with 10 ng/ml HSP60 after 5 h and 24 h in A7r5 cells (Fig. 3a) or after 24 h in AoSMCs (Fig. 3b). The Boyden chamber-based migration results were consistent with those of the MTT cell viability assay, showing that HSP60 has cytotoxic effects at high concentrations (Fig. 3c).

### ERK-induced the migration in HSP60-treated A7r5 cells

**Figure 4:**
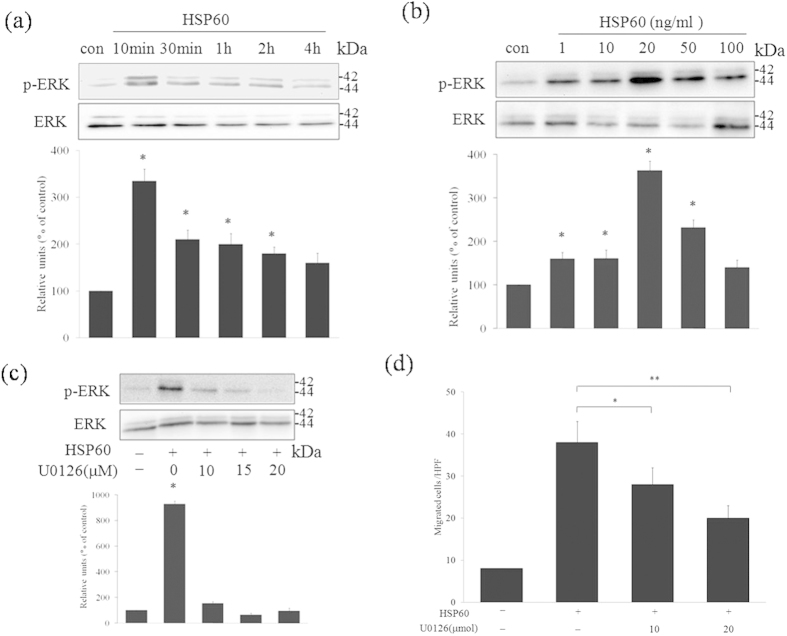
Relationship between the ERK MAPK signaling pathway and HSP60-induced cell migration. A7r5 cells were treated with 10 ng/ml HSP60 for 0–4 h (**a**). Cells were treated for 10 min with HSP60 at the indicated concentrations (**b**). Cells were pretreated with U0126 at the indicated concentrations for 30 min and then stimulated with 20 ng/ml HSP60. The cell lysates were analyzed by western blotting using an antibody against p-ERK. Equal protein loading was confirmed by detection using an ERK antibody (**c**). The numbers of migrated cells after exposure to HSP60 and the inhibitor were calculated (**d**). The results are expressed as the mean ± SD (*n *= 3). *p < 0.05, **p < 0.01 compared with the control. In (**a-c**), the full length blots were presented in Supplementary Figure S4 (**a-c**).

The relevance of the ERK and p38 MAPK signaling pathways in HSP60-induced migration of A7r5 cells was evaluated. As shown in Fig. 4a, stimulation by 20 ng/ml HSP60 induced a transient increase in the phosphorylation of ERK (p-ERK, Fig. 4a,b). Phosphorylation of p38 (p–p38) was not observed (data no shown). U0126, an inhibitor of ERK, caused a dose-dependent inhibition of p-ERK to basal levels (Fig. 4c). The inhibitor was found to block the HSP60-induced migration of A7r5 cells in a dose-dependent manner (Fig. 4d).

### TLR4-induced p-ERK activation and migration in HSP60-treated A7r5 cells

**Figure 5:**
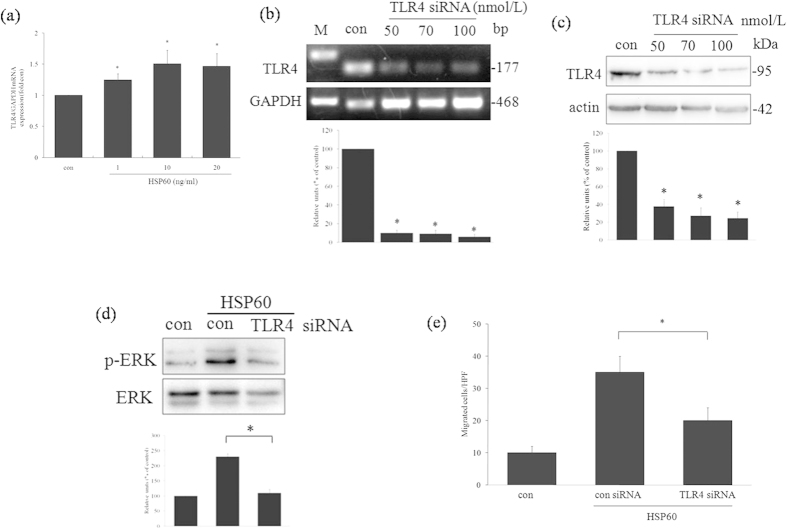
Relationship between TLR4 and HSP60-induced cell migration. A7r5 cells were treated with HSP60 at the indicated concentrations, and the expression of TLR4 mRNA was determined by real time RT-PCR (**a**). Cells were transfected with TLR4 siRNA, and the expression of TLR4 mRNA and protein was determined by RT-PCR (**b**) and western blotting (**c**). Cells were transfected with TLR4 siRNA, and the expression of p-ERK was analyzed by western blotting. Equal protein was confirmed by blotting against an ERK antibody (**d**). The numbers of migrated cells after treatment with HSP60 and after transfection with TLR4 siRNA were calculated (**e**). The results are expressed as the means ± SD (*n *= 3). *p < 0.05 compared with the control. In (**b-d**), the full length blots were presented in Supplementary Figure S5 (**b-d**).

Subsequently, the relevance of Toll-like receptors in the HSP60-induced migration of A7r5 cells was investigated. Real-time PCR data showed that exposure to HSP60 induced the expression of TLR4 (Fig. 5a). However, TLR2 expression was not induced (data not shown). To confirm the role of TLR4 in HSP60-stimulated A7r5 cell migration, siRNA technology was employed. Knockdown of TLR4 by siRNA specifically reduced the expression of TLR4 in A7r5 cells (Fig. 5b,c), which, in turn, blocked the expression of p-ERK (Fig. 5d) and decreased the number of cells that migrated in response to HSP60 (Fig. 5e).

### HSP60-induced stress fiber reconstitution in A7r5 cells

**Figure 6:**
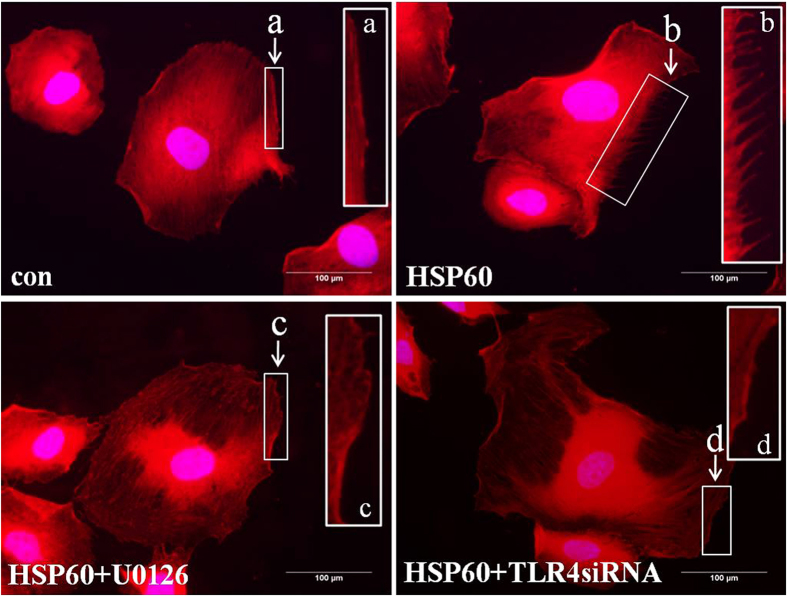
The effects of TLR4siRNA and U0126 on stress fiber formation after exposure to HSP60. A7r5 cells were transfected with TLR4 siRNA or exposed to U0126 after treatment with HSP60. Immunofluorescence labeling was achieved with phalloidin to image the actin fibers. The scale bar is 100 μm. A higher magnification is shown for stress fibers (**a**–**d**).

To further investigate the effects of HSP60, the reconstitution of stress fibers in A7r5 cells was studied by staining with tetramethyl rhodamine iso-thiocyanate (TRITC)-conjugated F-actin antibody after treatment with HSP60 with or without pretreatment with TLR4 siRNA and U0126. The formation of stress fibers was observed to be stimulated upon exposure to HSP60. However, pretreatment with TLR4 siRNA or U0126 was able to block the HSP60-induced formation of stress fibers (Fig. 6). These data support the hypothesis that HSP60 stimulates the migration of VSMCs through TLR4 and the ERK MAPK signaling pathway.

### HSP60 induced IL-8 secretion in A7r5 cells

**Figure 7:**
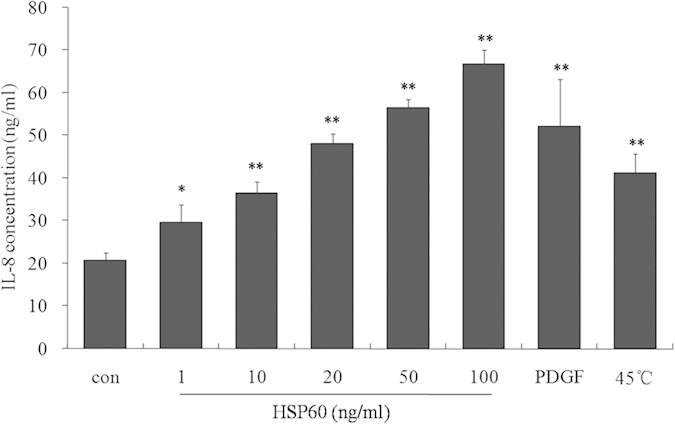
HSP60 induces IL-8 production in VSMCs. A7r5 cells were treated with HSP60 at the indicated concentrations, and IL-8 release was measured in the cell conditioned media. The results are expressed as the means ± SD (*n *= 3). *p < 0.05, **p < 0.01 compared with the control.

IL-8 has been known to play an important role in monocyte migration into the subendothelial space in the early phase of atherosclerosis. In addition, elevated levels of IL-8 are reportedly associated with an increased risk of future coronary artery disease^21^,^22^. We, therefore, investigated whether HSP60 induces the expression of IL-8 in VSMCs. Our data showed that HSP60 induced the expression of IL-8 in a dose-dependent manner, and similar effects of PDGF-BB and treatment by heat shock (45 °C) were observed in A7r5 cells (Fig. 7).

## Discussion

HSP60 is expressed at high levels in atherosclerotic lesions in humans, rabbits, and apolipoprotein E-deficient (APOE^−/−^) mice^23^. Kleindients *et al*. demonstrated that HSP60 is expressed in the endothelium, smooth muscle cells, and mononuclear cells of carotid and aortic specimens in humans^24^. In addition, HSP65 staining was found to be significantly increased in atherosclerotic lesions in rabbits^25^. In APOE^−/−^ mice, HSP60 is reported to be temporarily expressed on all major cell types in lesion-prone sites during the process of atherogenesis^26^. In this study, we investigated HSP60 expression in the aorta of mouse models of cardiovascular disease. In AS mice, our data showed that HSP60 was overexpressed at both the mRNA and protein levels in the thoracic aorta and atherosclerotic plaques. The same phenomenon was found in HFD mice. These results further support the important role of HSP60 in the development of atherosclerosis. Therefore, we investigated the molecular mechanisms underlying the role of HSP60 in atherosclerosis.

**Figure 8:**
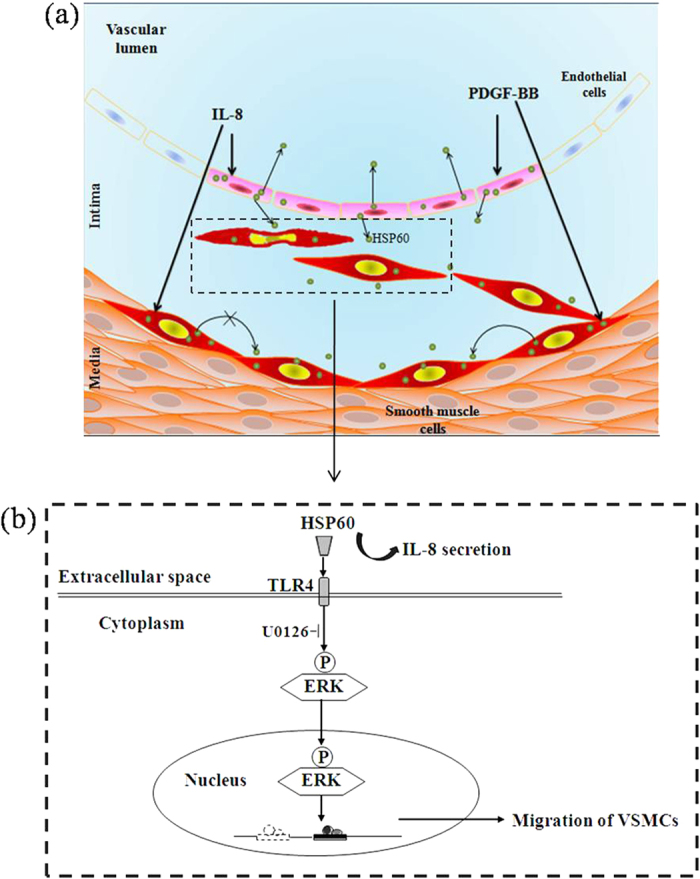
Overview the HSP60 secretion and expression in the blood vessel walls (a) and proposed signaling pathways in HSP60-regulated VSMC migration (b).

The quest to identify the molecular mechanisms of HSP expression and signaling was initiated a decade ago by Xu^23^. In subsequent years, several groups have contributed to this topic, but many published results have been rather controversial. Hirono *et al*. demonstrated that *Chlamydia pneumonia* infection induces endogenous HSP60 expression in VSMCs, but not in HUVECs. It was also demonstrated that endogenous HSP60 can stimulate VSMC proliferation. Sasu *et al*. reported that *C. pneumonia* and Chlamydia HSP60 stimulate proliferation of human VSMCs. Grundtman *et al*. hypothesized that endothelial cells at sites prone to atherosclerotic plaque formation are especially susceptible to this sequence of events^3^. Our previous studies have shown that PDGF-BB and IL-8 can stimulate the migration and proliferation of VSMCs, events that are key to the processes of atherosclerotic plaque formation^16^,^17^,^18^. We also investigated the relationship between PDGF-BB or IL-8 and HSP60 in endothelial cells and SMCs of blood vessels. Our data suggest that: 1) PDGF-BB can stimulate expression of HSP60, which is released and accumulates outside of HUVECs and VSMCs; 2) IL-8 only can stimulate the secretion of HSP60 in HUVECs; and 3) PDGF-BB and IL-8 can promote the HSP60 expression in HUVECs and VSMCs (Figs 2 and 8a). These results suggest that HSP60 is an important molecular regulator in the blood vessel wall. As endothelial cells and VSMCs release HSP60 into the extracellular space upon stimulation, we next investigated whether HSP60 can affect VSMC function. We used recombinant HSP60 from a rat or human source to stimulate A7r5 cells and human AoSMCs. We found that recombinant HSP60 was able to induce the migration and proliferation of VSMCs. To our knowledge, this is the first report showing that recombinant HSP60 directly stimulates VSMC migration. We used the recombinant HSP60 of the same species to imitate the endogenous protein that induced cell proliferation, and our findings are consistent with the results presented by Hirono *et al*.^27^.

Microbial antigens may interact with multiple intracellular signaling pathways. In a previous study, we indicated that the HSP60 protein of *Helicobacter pylori* induces IL-8 expression via TLR2 and the MAPK pathway^28^. Sasu *et al*. confirmed that chlamydial HSP60 is a potent inducer of human VSMC proliferation and that these effects are mediated^14^, at least in part, by rapid TLR4-induced activation of ERK MAPK signaling^15^. TLRs and the ERK signaling pathway may also be involved in the regulation of HSP60-mediated cell migration and proliferation. Our data showed that p-ERK is already activated 10 minutes after exposure to HSP60 and that U0126 (an inhibitor of ERK) decreased HSP-mediated VSMC migration. TLR4 is activated after HSP60 stimulation but TLR2 is not. TLR4 siRNA decreased p-ERK expression as well as HSP60-mediated cell migration. In this study, we demonstrated that HSP60 stimulates the migration of VSMCs via TLR4 and ERK MAPK activation.

Atherosclerosis is an inflammatory disease, and the expression of HSP60 by VSMCs may be related to the secretion of inflammatory cytokines. Our results showed that IL-8 expression by VSMCs was induced by HSP60, PDGF-BB, or exposure to heat (45 °C). Through this research, we were able to demonstrate that the interactions among HSP60, PDGF-BB, and IL8 increased cell migration. It is difficult to determine which signaling molecule is most essential. Both HSP60 and PDGF-BB can induce IL-8 expression; therefore, we presume that IL-8 plays a major stimulatory role in VSMC migration and that IL-8 expression is the signal for HSP60 overexpression.

The results of the present study provide a partial understanding of the function of HSP60 in atherosclerosis development: 1) both PDGF-BB and IL-8, under stress conditions, can activate HSP60 expression and release from endothelial cells and SMCs (Fig. 8a); 2) recombinant HSP60 stimulates the migration of VSMCs via TLR4 and ERK MAPK activation (Fig. 8b); and 3) HSP60 expression is a potent ‘danger signal’ to the immune system to generate IL-8, which assists in the management of an infection or disease.

## Methods

### Chemicals

Recombinant HSP60 was purchased from Assay Designs (Enzo Life Sciences, Switzerland). The HSP60 polyclonal antibody (15282-1-AP) was obtained from Proteintech (Proteintech Group, Inc., USA). ERK (BS1112), phospho-ERK (BS5016), and CD31 antibody (BS1574) were obtained from Bioworld Technology (St Paul, MN, USA). The ERK inhibitor U0126 and actin antibody were purchased from Cell Signaling Technology (USA). The TLR4 siRNA was obtained from Thermo (USA). PDGF-BB, IL-8, fluorescein isothiocyanate (FITC)-conjugated anti-α-smooth muscle actin antibody (F3777) and phalloidin-tetramethyl rhodamine B isothiocyanate (P1951) were obtained from Sigma-Aldrich (St Louis, MO, USA).

### Animal experiments

This study was conducted in accordance with the Guide for the Care and Use of Laboratory Animals published by the US National Institutes of Health (8^th^ edition, 2011).

HFD model: Male BALB/c mice (6 weeks old) were obtained from the Dalian Medical University Laboratory Animal Center. The mice were randomly divided into the control diet group (n = 6, fed standard chow) and the HFD group (n = 12). Those in the HFD group were fed a diet consisting of 78% common chow, 10% lard oil, 10% yolk powder, 1% cholesterol, and 0.2% bile salt from pig^20^.

AS model: APOE^−/−^ mice and wild-type (WT) control (C57BL/6J) mice were originally purchased from Beijing Vital River Laboratory Animal Technology Co., Ltd. WT control mice were fed standard chow (n = 6), and APOE^−/−^ mice were fed with a hign fat diet (n = 12).

After 12 weeks (in the AS model) or 8 months (in the HFD model), mouse aortas were excised, snap frozen in liquid nitrogen, and stored at −80 °C for reverse transcriptase polymerase chain reaction (RT-PCR) analysis. Samples of the aortic root were fixed in 4% paraformaldehyde and embedded in paraffin using standard procedures for subsequent immunohistochemical analyses. The animal protocol was approved by the local research ethics review board of the Animal Ethics Committee of Dalian Medical University.

### RT-PCR analysis

The total RNA was initially extracted using RNAiso Plus (TaKaRa, Japan) according to the manufacturer’s protocol. All RNA samples were digested with RNase-free DNase I (Invitrogen, USA). RNA from each sample (300 ng) was reverse transcribed to cDNA with random 9-mers or without random 9-mers as a negative control. cDNA was amplified in a total volume of 50 μl using the TaKaRa RNA PCR Kit (TaKaRa, Japan).

The primers used for real-time PCR were as follows: glyceraldehyde-3-phosphate dehydrogenase (GAPDH): 5′-TGTGTCCGTCGTGGATCTG-3′ (sense) and 5′-TTGCTGTTGAAGTCGCAGGAG-3′ (antisense); HSP60: 5′-TGATGTTGGCTGTGGATGCT-3′ (sense) and 5′-GACACCCTTTCTTCCAACCTTT-3′ (antisense); TLR2: 5′-GGCCACAGGACTCAAGAGCA-3′ (sense) and 5′-AGAGGCCTATCACAGCCATCAAG-3′ (antisense); and TLR4: 5′-CTCACAACTTCAGTGGCTGGATTTA-3′ (sense) and 5′-GTCTCCACAGCCACCAGATTCTC-3′ (antisense). The real-time PCR cycling parameters (30 cycles) were set as follows: denaturation (95 °C, 30 sec), annealing (56 °C, 30 sec), and extension (72 °C, 15 sec).

The primers used in PCR were as follows: GAPDH: 5′-CCATCACTGCCACTCAGAAGAC-3′ (sense) and 5′-TACTCCTTGGAGGCCATGTAGG-3′ (antisense), which yielded a 468-bp fragment; and TLR4: 5′-CTCACAACTTCAGTGGCTGGATTTA-3′ (sense) and 5′-GTCTCCACAGCCACCAGATTCTC-3′ (antisense), which yielded a 177-bp fragment. The PCR cycling parameters (35 cycles) were set as follows: denaturation (94 °C, 30 sec), annealing (56 °C, 30 sec), and extension (72 °C, 30 sec for GAPDH or 11 sec for TLR4). Equal amounts of PCR products were electrophoresed on 2% agarose gels and visualized by ethidium bromide staining. The specific bands of PCR products were analyzed using an Image-Pro Plus 6.0 system with GAPDH used as the internal control for normalization.

### Immunohistochemical and immunofluorescence staining for tissues

The aortas were processed into 3-μm-thick serial sections and stained with hematoxylin/eosin to detect the development of atherosclerotic lesions. Additional aortic sections were used for immunohistochemical staining to detect HSP60 or immunofluorescence staining for HSP60, α-smooth muscle actin, or CD31. Briefly, Paraffin sections were deparaffinized with dimethylbenzene and ethanol, antigen was retrieved by incubation in 0.01 M citrate buffer (pH 6.0) at 95 °C for 20 min. The samples were rinsed three times with PBS, incubated for 15 min at room temperature with a protein-blocking solution of 5% fetal bovine serum in PBS (pH 7.5), washed three times with PBS, and then incubated with the primary antibodies (HSP60 antibody, 1:100; FITC-conjugated α-smooth muscle actin antibody, 1:100; and CD31 antibody, 1:100) at 4 °C overnight. The samples were then rinsed three times with PBS and incubated with the biotinylated secondary antibody or fluorochrome-conjugated secondary antibody for 40 min at 37 °C. Finally, the specimens were stained with DAPI. Digital images were taken using a fluorescence microscope (BX-51, TR32000, Olympus, Tokyo, Japan) with a charge-coupled device system. The results were quantitated using the Image-Pro 6.0 Microsoft program (MediaCybernetics, Bethesda, MD).

### Cell culture

The A7r5 vascular SMC line, originally derived from embryonic rat aorta^29^, was purchased from the American Type Culture Collection (ATCC, Manassas, VA, USA). The cells were maintained in Dulbecco’s modified Eagle’s medium (DMEM) supplemented with 10% fetal bovine serum (FBS) and antibiotics. Human AoSMCs, originally derived from human aortic SMCs, were purchased from ATCC and grown in DMEM supplemented with 10% FBS, 2 ng/mL human basic fibroblast growth factor, 0.5 ng/mL human epidermal growth factor, 5 μg/mL insulin, and antibiotics. HUVECs were obtained from Institute of Biochemistry and Cell Biology, CAS (Shanghai, China) and cultured in DMEM medium containing 10% FBS and antibiotics. The cells were maintained at 37 °C in a CO_2_ incubator (in a humidified atmosphere of 5% CO_2_ and 95% air). The cells were grown to 80–90% confluence. Quiescence was achieved by serum starvation overnight.

### Western blotting

HUVECs were plated in replicates at a density of 2 × 10^5^ per well (VSMCs were plated at 1 × 10^5^ cells per well). The cells were treated with HSP60, PDGF-BB, or IL-8. Following treatment, conditioned media was centrifuged at 1,000 × g for 5 minutes to remove any non-adherent or dead cells. The conditioned media was collected the total protein concentration of the cells in each well was determined using 150 μl RIPA Lysis Buffer (Beyotime) after centrifugation at 15,000 × g for 20 minutes. 8 μl supernatant containing the cellular protein or 20 μl conditioned media was loaded onto a 12% SDS-PAGE gel and blotted onto a polyvinylidene difluoride membrane (Millipore, Billerica, MA, USA). The membranes were washed with Tris-buffered saline (TBS) and blocked with 5% (w/v) non-fat milk in TBS-Tween 20 (1%, v/v, TBST) for 1 h at room temperature. After three washes of 5 min each with TBST, the membranes were incubated with a rabbit actin polyclonal antibody, ERK antibody, and a phospho-ERK antibody in TBST with 5% (w/v) bovine serum albumin (BSA) overnight at 4 °C. After three washes with TBST, the blots were incubated with horseradish peroxidase (HRP)-linked anti-rabbit immunoglobulin (Ig)G antibody (Jackson ImmunoResearch, USA) for 1 h at room temperature. The bands were visualized by enhanced chemiluminescence (ECL) detection after Western blotting (the detection reagents were obtained from Amersham Bioscience, Piscataway, NJ, USA). Quantitative analysis was performed using a Scion Image System (Frederick, MD, USA). The band intensities were analyzed using Image Gauge^®^ Ver. 4.0 software (Fuji Film, Tokyo, Japan).

### Migration assay

Cell migration was evaluated using a Boyden chamber (Neuroprobe, Gaithersburg, MD, USA) as previously described (Zhao *et al*., 2012). In all of the experiments, collagen-coated polycarbonate filters (pore size, 8 μm) were used. The cells (3 × 10^5^ cells/well) suspended in serum-free DMEM containing 0.5% bovine serum albumin (DMEM-BSA) were loaded into the upper wells of the chamber. The lower wells were filled with DMEM-BSA containing HSP60. After a 5 h or 24 h incubation at 37 °C, the cells that had migrated to the lower surface of the filter were fixed in methanol, stained with Giemsa solution, and observed using a light microscope. Each condition was tested in quadruplicate, and the numbers of cells in randomly chosen high-power fields (×200 magnification) were counted in all wells. In certain experiments, cells were pretreated with TLR4 siRNA and the MAPK/ERK kinase 1 (MEK1) inhibitor U0126 for 1 h. The migration assay was performed in at least three independent experiments.

### MTT assay

To evaluate cell viability, 5 × 10^3^ cells were plated in each well of a flat-bottom 96-well culture plate, incubated in 100 μL DMEM medium for 24 h, and treated according to the described experimental conditions. After treatment, methylthiazolyl tetrazolium (MTT) was added to each well to a final concentration of 0.5 mg/mL and incubated for 4 h at 37 °C in a humidified incubator containing 5% CO_2_. To dissolve the cells, we used 100 μL DMSO and measured the absorbance of the resulting solutions at 570 nm using a microplate reader (Thermo Scientific, USA). The results are presented as a percentage of cell viability (the optical density [OD] of the experiment samples/the OD of control group).

### Transfection of siRNAs

The sequences of TLR4 siRNA were pre-designed and synthesized by Thermo Fisher Scientific. The negative control siRNA was purchased from TaKaRa Biotechnology. A7r5 cells were transfected using Lipofectamine 2000 (Invitrogen) according to the manufacturer’s instructions.

### Immunofluorescence assay

The A7r5 cells transfected with TLR4 siRNA or pretreated with U0126 were grown on sterile glass cover slips. The cells were washed in ice-cold PBS and fixed for 20 min at room temperature in 4% paraformaldehyde. After fixation, the cells were permeabilized with 0.1% TritonX-100 for 10 min and blocked with 5% BSA in PBS for 20 min. The cells were then incubated with rabbit anti-F-actin antibody conjugated with TRITC Sigma-Aldrich) for 40 min. Chromatin was stained with DAPI, and cells were mounted onto slides for photography using a fluorescence microscope (BX-51, TR32000, Olympus, Tokyo, Japan) with a charge-coupled device system.

### IL-8 secretion from cells

The A7r5 cells were stimulated with HSP60 at the specified concentrations, along with PDGF-BB at 37 °C or exposure to heat-shock treatment at 45 °C. The conditioned media and cells were separated by centrifugation at 300 × g and stored at −80 °C until assayed. The conditioned media were assayed for IL-8 production using an enzyme-linked immunosorbent assay (IL-8 ELISA kit, BD, USA).

### Statistical analyses

Data were analyzed using SPSS (Statistical Product and Service Solutions). All data are presented as mean ± standard error (SE). For comparisons between two groups the unpaired Student’s t test was employed. For comparisons among multiple (≥3) groups, one way analysis of variance (ANOVA) was used followed by the Student-Newman-Keuls test and differences assessed with Dunnett’s posttest. Statistical significance was defined as p ≤ 0.05.

## Additional Information

**How to cite this article**: Zhao, Y. *et al*. Heat shock protein 60 stimulates the migration of vascular smooth muscle cells via Toll-like receptor 4 and ERK MAPK activation. *Sci. Rep*. **5**, 15352; doi: 10.1038/srep15352 (2015).

## Supplementary Material

Supplementary Information
