# Supplementary material for: Heat shock protein 60 stimulates the migration of vascular smooth muscle cells via Toll-like receptor 4 and ERK MAPK activation
Source: Sci Rep. 2015 Oct 19;5:15352. doi: 10.1038/srep15352 (PMC4609986; doi:10.1038/srep15352)
Supplement: Supplementary Information [file srep15352-s1.doc]

***Supplementary Information***

**Heat shock protein 60 stimulates the migration of vascular smooth muscle cells via Toll-like receptor 4 and ERK MAPK activation**

Ying Zhao^1,2^, ChenXu Zhang^2^, XuGe Wei^2^, Pei Li^2^, Ying Cui^1,2^, YuanHua Qin^2^, XiaoQing Wei^1^, MinLi Jin^1^, Kazuhiro Kohama^3^ & Ying Gao^1,2﹡^

^1^Liaoning Provincial Core Lab of Medical Molecular Biology, Dalian Medical University, Dalian, 116044, China

^2^Department of Biochemistry and Molecular Biology, Dalian Medical University, Dalian, 116044, China

^3^Research Institute of Pharmaceutical Sciences, Musashino University, Nishitokyo, Tokyo, 2028585, Japan

Correspondence and requests for materials should be addressed to Y.G. (gaoying822@hotmail.com)

**
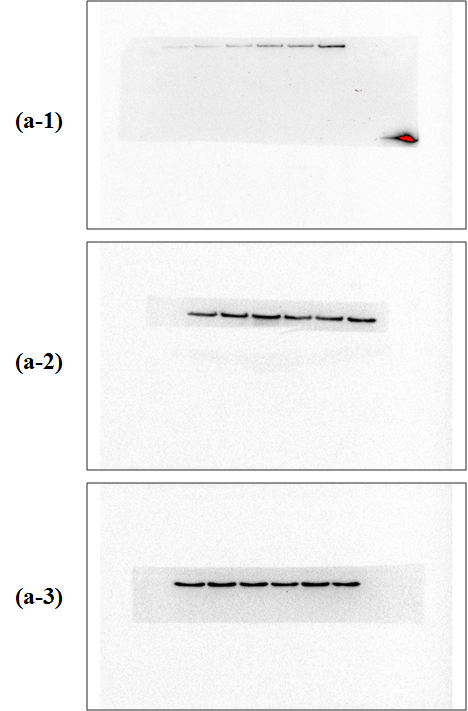
**

**Figure S2a∣Full-length blots.**

**
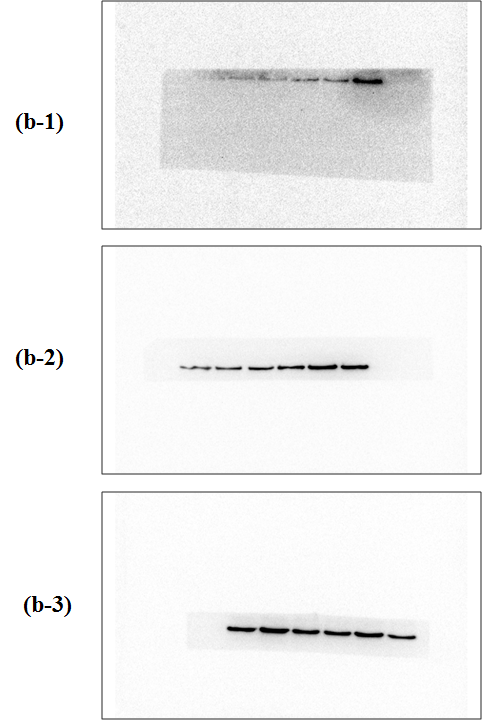
**

**Figure S2b∣Full-length blots.**

**
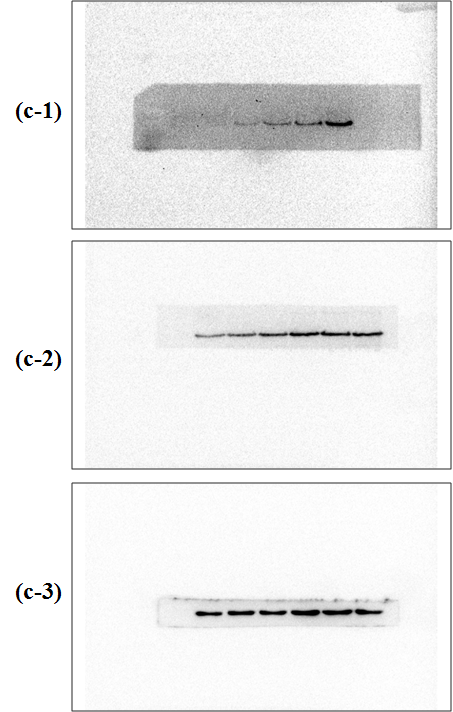
**

**Figure S2c∣Full-length blots**


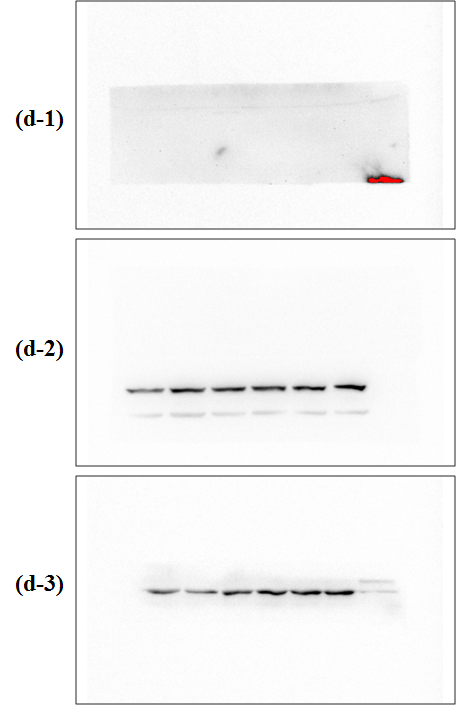


**Figure S2d∣Full-length blots.**


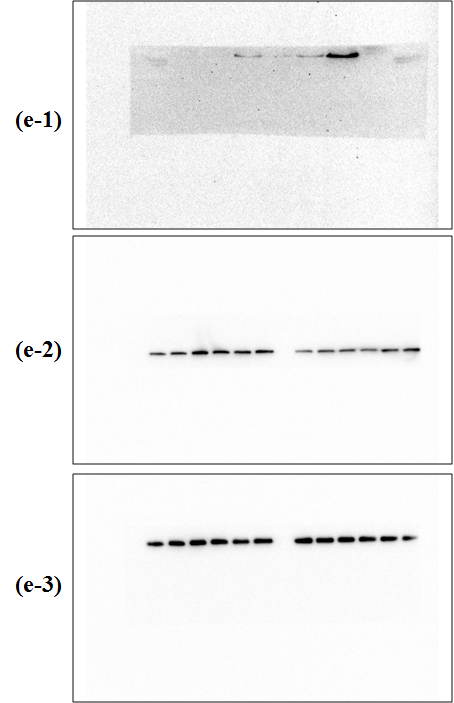


**Figure S2e∣Full-length blot.**


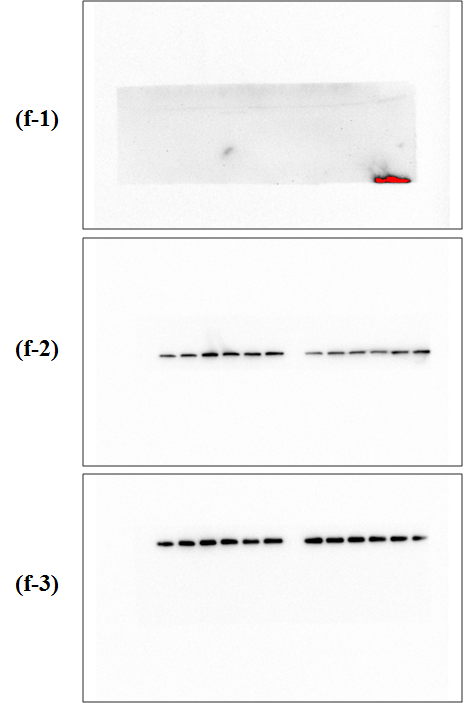


**Figure S2f∣Full-length blots.**

**
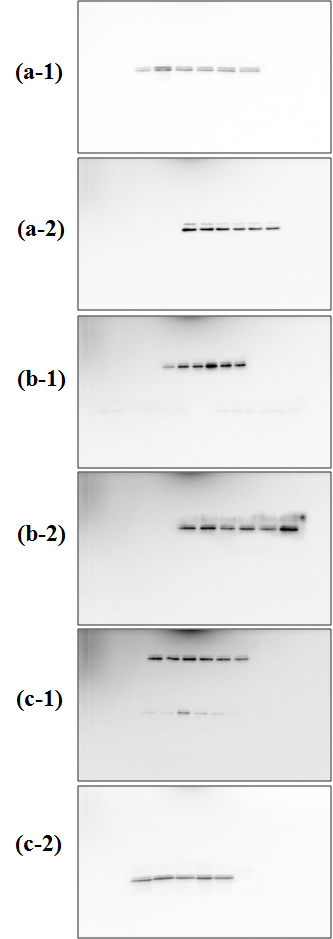
**

**Figure S4∣Full-length blots.**

**
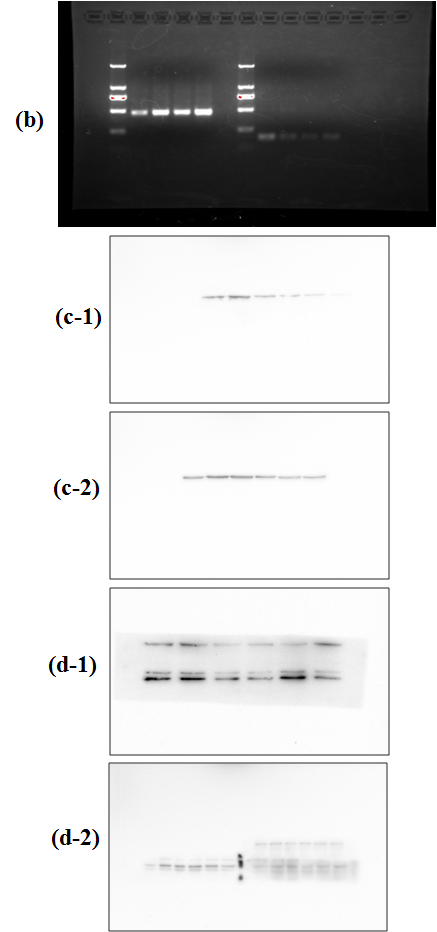
**

**Figure S5∣Full-length gels and blots.**
